# Effect of nanoparticles on gouty arthritis: a systematic review and meta-analysis

**DOI:** 10.1186/s12891-023-06186-3

**Published:** 2023-02-14

**Authors:** Ruiting Zhu, Yirou Niu, Wei Zhou, Saikun Wang, Jing Mao, Yingze Guo, Yangyang Lei, Xuance Xiong, Yingzhi Li, Lirong Guo

**Affiliations:** 1 School of Nursing, Jilin University, Changchun, 130021 Jilin China; 2 The First Hospital of Jilin University, Changchun, 130021 Jilin China; 3 Medical College, Beihua University, Jilin, 132013 Jilin China; 4 Orthpoeadic Medical Center, Jilin University Second Hospital, Changchun, 130041 Jilin China

**Keywords:** Nanoparticles, Uric acid, Gouty arthritis, Animal experiment, Meta-analysis

## Abstract

**Objective:**

The purpose of this study was to explore the effects of nanoparticles on gouty arthritis, and to provide evidence for the preclinical application of nanoparticles in gouty arthritis and ideas for nanomedicine improvement for nanoparticle researchers.

**Methods:**

Five databases including the Cochrane Library, PubMed, Scopus, Web of Science, and Embase were searched for eligible studies until April 2022. The quality of the selected studies was assessed by SYRCLE’s risk of bias (RoB) tool, and the random-effects model was used to calculate the overall effect sizes of weighted mean differences (WMD).

**Results:**

Ten studies met the inclusion criteria. Results showed that nanoparticles were effective in reducing uric acid levels (WMD: -4.91; 95% confidence interval (CI): − 5.41 to − 4.41; *p* < 0.001), but were not better than allopurinol (WMD: -0.20; 95% CI: − 0.42 to 0.02; *p* = 0.099). It was worth noting that the nanoparticles were safer than allopurinol. Subgroup analyses indicated that nanoparticle encapsulated substance, animal species, nanoparticle dosage, animal quantity, and animal gender were all sources of heterogeneity.

**Conclusion:**

The nanoparticles are safe medications for gouty arthritis which can effectively reduce uric acid levels in rodents. Although the results are still uncertain, it is expected to have certain clinical application value. The nanoparticles may be the preclinical medications for gouty arthritis in the future.

**Supplementary Information:**

The online version contains supplementary material available at 10.1186/s12891-023-06186-3.

## Introduction

Gout is a form of inflammatory arthritis caused by the chronic deposition of monosodium urate (MSU) crystals [1]. MSU deposition is caused by an increase in blood uric acid levels due to insufficient excretion or excessive secretion of uric acid [2]. The incidence and prevalence of gouty arthritis are increasing due to unhealthy lifestyles and/or diets [3]. According to epidemiological studies, the incidence rate of gouty arthritis was 0.59‰-2.89‰ per year, and the prevalence was 0.02% to 6.8% worldwide [4]. Gouty arthritis can seriously affect people’s daily life. Currently, medical therapy, surgery, and rehabilitation exercise are routine clinical treatments for gouty arthritis. Furthermore, allopurinol is the most commonly used medication for gouty arthritis [5], allopurinol is a xanthine oxidase (XO) inhibitor, which can reduce uric acid production [6]. However, allopurinol has some unavoidable side effects, such as gastrointestinal irritation, cutaneous reactions, bone marrow suppression, hypersensitivity reactions, and renal toxicity [7–9]. Therefore, it is necessary to develop some new medications with fewer side effects to reduce uric acid levels in patients with gouty arthritis.

In recent years, nanoparticles have been applied in malignant perivascular epithelioid cell tumor [10], hepatic fibrosis [11], inflammatory bowel diseases [12], ankylosing spondylitis [13], and other inflammatory diseases. Gouty arthritis as an inflammatory arthritis may be alleviated by nanoparticles. At present, some researchers have applied nanoparticles to treat gouty arthritis in animal experiments and proved its efficacy on reduction in uric acid levels. Compared with allopurinol, nanoparticles can improve the biocompatibility and bioavailability of the substance [14], and reduce the toxicity and side effects of substances [15].

Nowadays, the encapsulated substances in nanoparticles most commonly used by researchers mainly include three categories: metals oxides, non-metals and biologically active substances. Metal oxide nanoparticles include copper oxide nanoparticles (CuO-NPs), zinc oxide nanoparticles (ZnO-NPs), and iron oxide nanoparticles (FeO-NPs). Among them, CuO-NPs can reduce oxidative stress [16], ZnO-NPs can inhibit the synthesis of mRNA expression of inflammatory cytokines [17], FeO-NPs can reduce inflammation [18]. Moreover, for non-metallic substances encapsulated in nanoparticles, *Puerariae* *lobatae* Radix (PLR) has analgesic and anti-inflammatory effects [19], Aurantii fructus immaturus (AFI) is often used in the treatment of inflammatory and metabolic diseases [20], turmeric can relieve inflammation and pain [21], and Ginsenoside Rb1 (GsRb1) can reduce inflammatory cytokines and oxidative stress levels [22]. In addition, among biologically active substances, IL-1 receptor antagonist (IL-1Ra) has a rapid anti-inflammatory effect [23], and uricase can reduce uric acid levels by oxidizing uric acid to allantoin and hydrogen peroxide [24]. To sum up, these substances are often encapsulated in nanoparticles by researchers to treat gouty arthritis.

Nevertheless, the effects of nanoparticles on gouty arthritis are inconclusive. Wang X et al. [25] found that PLR-CDs reduced uric acid levels by inhibiting XO. Sohail MF et al. [21] found that turmeric nanoparticles (T-NPs) were rich in polyphenols to achieve antioxidant effects. Kiyani et al. [17] found that ZnO-NPs could effectively inhibit the formation of uric acid. Therefore, the aims of this study were to explore the effects of nanoparticles on gouty arthritis and provide evidence for the preclinical application of nanoparticles in gouty arthritis.

## Methods

### Search strategy

This systematic review and meta-analysis followed the Cochrane Handbook for Systematic Reviews of Interventions [26] and the PRISMA (Preferred Reporting Items for Systematic Review and Meta-analyses guidelines) [27]. PRISMA 2020 Checklist was shown in Supplementary Table S1. The study protocol was registered in PROSPERO (Number: CRD42021277015). Eligible studies that evaluated the effectiveness of nanoparticles on gouty arthritis were searched in the Cochrane Library, PubMed, Scopus, Web of Science, and Embase from all published studies until April 2022. In addition, we searched the reference lists of the included studies and identified other relevant studies. Two reviewers (WZ and SKW) had independently screened the retrieved articles to identify potentially eligible studies based on inclusion and exclusion criteria. A related search strategy was created using various combinations of predefined search terms (arthritis gouty, gouty arthritis, arthritides, gouty, gouty arthritides, synovial joints, gout, uric acid, hyperuricemia, uric acid crystals; nanostructures, nanostructure, nanostructured materials, material, nanostructured, materials, nanostructured, nanostructured material, nanomaterials, and nanomaterial) and Boolean search terms (AND, OR, and NOT), which were entered as search terms into each database. Details of the search strategy were shown in Supplementary Table S2.

### Inclusion and exclusion criteria

The included studies met the following criteria: 1) animal experiment; 2) successfully established a gouty arthritis model; 3) the intervention was nanoparticle; 4) the control group was commonly used medicine for gouty arthritis treatment, such as allopurinol and indomethacin; 5) the outcomes included the serum uric acid level and/ or joint swelling degree; 6) published in English.

The excluded studies met the following criteria: 1) notes, comments, reviews, and editorials; 2) duplicated studies; 3) unclear outcome indicators.

### Quality assessment

The quality of each included study was assessed independently by two reviewers (JM and YZG) using SYRCLE’s risk of bias (RoB) tool [28]. A consensus-oriented discussion or a third reviewer (YRN) made the final decision when a conflict occurred. The quality of studies was evaluated according to the following aspects: sequence generation (selection bias), baseline characteristics (selection bias), allocation concealment (selection bias), random housing (performance bias), blinding (performance bias), random outcome assessment (detection bias), blinding (detection bias), incomplete outcome data (attrition bias), selective outcome reporting (reporting bias), and other sources of bias. A high risk of bias was indicated by “no”, a low risk of bias was indicated by “yes”, and an unclear risk of bias was indicated by “unclear”. A study would be considered high quality if it had no more than one unclear risk. If a study had no more than three unclear risks or one high-risk, the quality would be considered medium. A study would be considered low quality if it had more than three unclear risks or two high-risks [29].

### Data extraction

Two reviewers (YYL and XCX) independently extracted the data and assessed their quality. Any cases of disagreement were arbitrated by a third reviewer (YRN). The information extracted from the studies included the first author, publication year, animal species, gender, quantity, weight, group, intervention, dosage, method of administration, control group, model group, and measured outcomes.

### Statistical analysis

Statistical analyses were performed using the Stata software (version12.0 SE; Stata Corp LP, College Station, TX, USA). The effect sizes of this meta-analysis were defined as the weighted mean difference (WMD) and the 95% confidence interval (CI). I^2^ statistics were used to assess the studies’ heterogeneity of the pooled results. An I^2^ value < 25% represented low heterogeneity, an I^2^ value 25% –50% represented medium heterogeneity, and an I^2^ value 50% –100% represented high heterogeneity. Because the heterogeneity of this study is high, and compared with fixed-effects model, the random-effects model is more conservative [30]. Therefore, a random-effects model was used for the meta-analysis. Since some studies contained more than two experimental groups, we included several experiment groups from one study and included them in the meta-analysis, and the sample sizes of their control groups were divided for analysis. We explored the source of heterogeneity through sensitivity and subgroup analyses. Subgroup analyses were performed according to nanoparticle dosage, number of animals, gender of animals, animal species, and encapsulation substance of nanoparticles. Leave-one-out sensitivity analyses were used to examine whether a single study affected the pooled effect size and to evaluate the source of heterogeneity. Funnel plots were generated, and Egger’s tests were performed to evaluate the possible publication bias. However, the detection effects of these two methods for publication bias were limited when the number of studies included is less than 10 [31]. Considering that both methods still have some validity, funnel plots and Egger’s test were performed to evaluate possible publication bias in the present study, but the results were for reference only. All the statistical tests were two-sided, and a *p*-value < 0.05 was considered statistically significant.

## Results

### Study selection

**Fig. 1:**
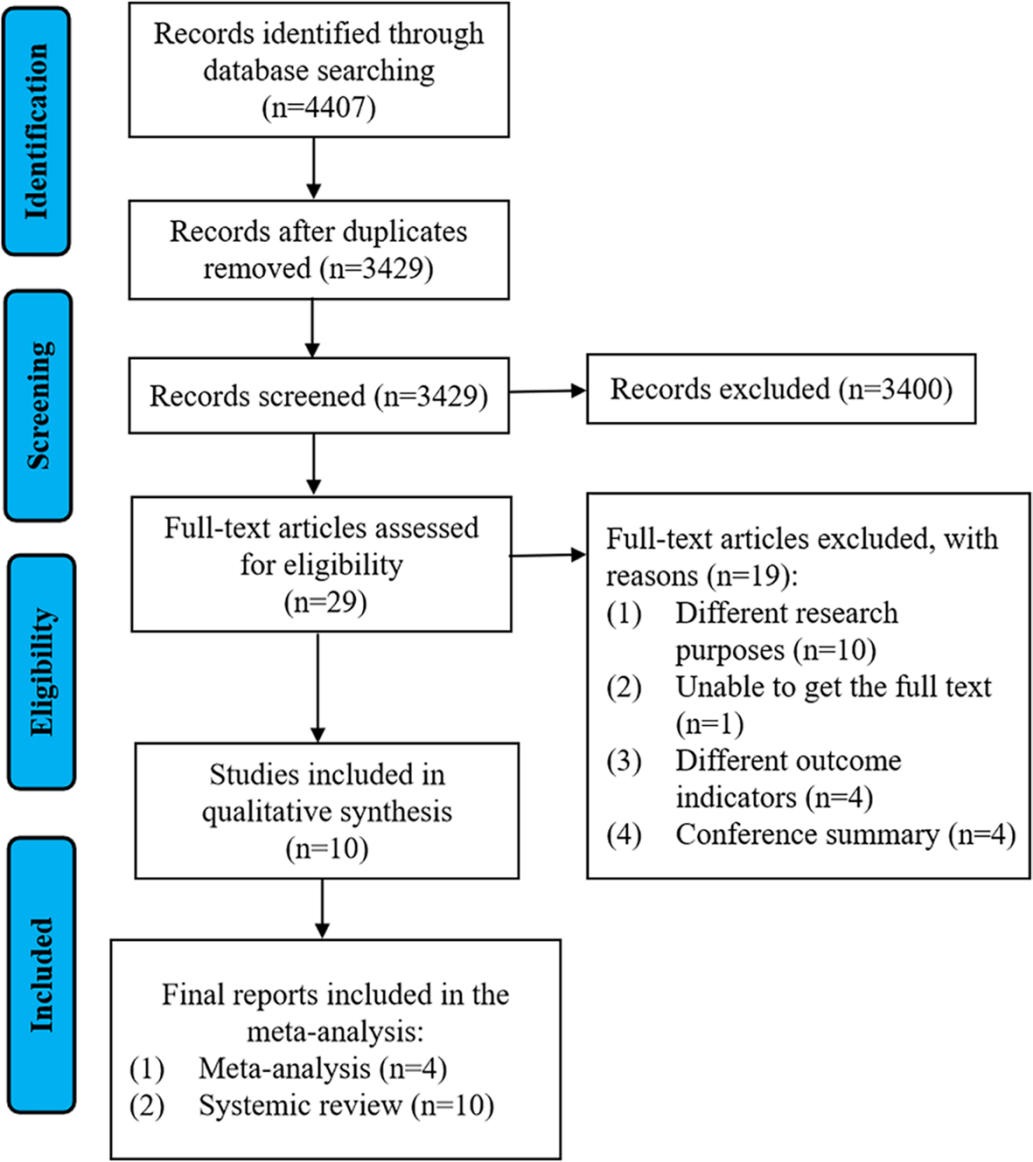
Flowing diagram of included studies selection process

Figure 1 showed the flow chart of the literature search. 4407 studies were identified and screened during the systematic search, 978 studies were excluded using the Endnote software (X9, Thomson Corporation, Connecticut, USA) for deduplicate removal. Then 3400 studies were excluded after screening the title and abstract, and 19 studies were excluded after full-text screening. Eventually, 10 studies [16, 17, 20–25, 32, 33] were included in the present systematic review, of which four studies [20, 21, 25, 32] provided specific data for integration and analysis for the meta-analysis.

### Characteristics of the included studies

All studies [16, 17, 20–25, 32, 33] that met the inclusion and exclusion criteria were published from 2019 to 2022 with 30 to 140 animals in each study. Four studies [16, 17, 32, 33] were on metal oxide (CuO, ZnO, and FeO), four studies [20–22, 25] were on non-metallic substances (PLR, AFI, turmeric, GsRb1), and two studies [23, 24] were on biologically active substances (IL-1Ra and uricase). Five studies [16, 17, 21, 32, 33] used BALB/c mice, four studies [20, 22, 23, 25] used Sprague Dawley (SD) rats, and only one study [24] used the Institute of Cancer Research (ICR) mice. Among the 10 studies included, five studies [20, 22–25] included only male animals, and the remaining five studies [16, 17, 21, 32, 33] included both male and female animals. All studies included in this study were quasi-experiments. Allopurinol was used as a control medication in seven studies [16, 17, 20, 21, 25, 32, 33], indomethacin was used as a control medication in two studies [22, 23], and one study [24] was a self-control before and after the intervention. The level of serum uric acid [17, 20, 21, 24, 25, 32, 33] and the degree of joint swelling [20, 22, 23, 25, 32] reflected the effect of nanoparticles on treating gouty arthritis. Kidney and liver function, blood lipids, and blood cells reflected the safety and side effects of nanoparticles. Table 1 showed the detailed information of the included studies.

**Table 1:** Main information extracted from included studies

Study	Year	Animal species	Gender	Quantity	Weight(g)	Group	Intervention	Dosage	Method of administration	Control group	Dosage	Model group	Measured outcomes
Kiyani MM; Butt MA	2021	BALB/c mice	F; M	36	25–30	6	CuO-NPs	5, 10, or 20 ppm	oral	Allopurinol	50 mg/kg	MSU	Histopathology
Wang S	2019	SD rats	M	48	200 ± 10	6	AFIC-CDs	2, 4, or 8 mg/kg	i.p.	Allopurinol	5 mg/kg	MSU	Uric acid levels serum; The swelling degree of joints
Kiyani MM; Moghul NB	2021	BALB/c mice	F; M	42	25–30	7	FeO-NPs	5, 10, or 20 ppm	oral	Allopurinol	50,100 mg/kg	MSU	Renal Function Test; Liver Function Test; Lipid Profile; Histopathology
Liu Y	2020	SD rats	M	40	180–220	5	GsRb1; nano-GsRb1	80 mg/kg	oral	IN	5 mg/kg	MSU	The swelling degree of joints; Histopathology
Kiyani MM; Butt MA	2019	BALB/c mice	F; M	140	25 ± 10	7	ZnO-NPs	5, 10, or 20 ppm	oral	Allopurinol	50 mg/kg	NPs control; MSU	Renal function test; Liver function test; Lipid profile; Blood count; Histopathology
Kiyani MM; Sohail MF	2019	BALB/c mice	F; M	36	25–30	6	T-NPs	5, 10, or 20 ppm	oral	Allopurinol	50 mg/kg	MSU	Liver function test; Lipid profile; Renal function test; Blood count; Histopathology
Kiyani MM; Rehman H	2020	BALB/c mice	F; M	48	25–30	8	CuO-NPs	5, 10, or 20 ppm	oral	Allopurinol	50,100 mg/kg	MSU; CuSO_4_ 40 mg/kg	Renal function test; Liver function test; Blood count; Lipid profile; Uric acid levels serum; The swelling degree of joints
Wang X	2019	SD rats	M	36	190–210	6	PLR-CDs	1, 2, or 4 mg/kg	oral	Allopurinol	5 mg/kg	OXO	The swelling degree of joints; Histopathology
Hao Y	2019	ICR mice	M	35	20–22	7	The uricase& HRP-CaHPO_4_ @HA MN	-	IM	The HA MN (IM); The uricase (I.v.); The uricase (s.c.); The uricase-CaHPO_4_@HA MN (IM)	1U/kg	OXO	Uric acid level serum; Histopathology; Blood count
Zhang J	2021	SD rats	M	30	-	5	IK-NPs	0.54 μmol/kg	s.c.	IN	2 mg/kg	MSU	The swelling degree of joints; Histopathology

*F* Females, *M* Males, *SD* Sprague–Dawley, *ICR* Institue of Cancer Research, *i.p.* Intraperitoneally, *i.v.* Intravenous, *s.c.* subcutaneous, *IM* Intramuscular injection, *OXO* Potassium oxonate, *IN* Indomethacin, *CuO-NPs* copper oxide nanoparticles, *AFIC-CDs* Aurantii fructus immaturus carbonisata-derived carbon dots, *FeO-NPs* iron oxide nanoparticles, *GsRb1* Ginsenoside Rb1, *ZnO-NPs* zinc oxide nanoparticles, *T-NPs* turmeric nanoparticles, *PLR-CDs* Puerariae lobatae Radix carbon dots, *IK-NPs* IL-1Ra bio-nanoparticles, *MSU* monosodium urate

### Risk of bias assessment

SYRCLE’s RoB tool was used to assess the risk of bias of included studies on animal experiments, the following results were obtained [34]: 100% of the included studies reported baseline characteristics, none of the studies provided details about sequence generation or allocation concealment, 100% reported information about random housing, 100% showed a low risk of bias in performance blinding, 10.0% of the included studies showed an unclear risk of bias in random outcome assessment, 70.0% showed a low risk of bias in detection blinding, 80.0% of studies showed a low risk of bias in incomplete outcome data, 100% showed a low risk of bias in selective outcome reporting, and none of the included studies reported other sources of bias.

The evaluation results of the studies showed a higher risk of bias. Among the 10 studies, nine studies [16, 17, 21–25, 32, 33] were of medium quality studies, and one study [20] was of low quality. Results of the studies using the SYRCLE’s risk of bias tool were shown in Table 2 and Supplementary Fig. S1.

**Table 2:** SYRCLE’s risk of bias tool

Author/Year	Baseline Characteristics	Sequence Generation	Allocation Concealment	Random Housing	Blinding (Performance)	Random Outcome Assessment	Blinding (Detection)	Incomplete Outcome Data	Selective Outcome Reporting	Other Sources of Bias
Kiyani MM; Butt MA, 2021 [16]	+	?	?	+	+	+	+	+	+	+
Wang S, 2019 [20]	+	?	?	+	+	+	+	-	+	+
Kiyani MM; Moghul NB, 2021 [33]	+	?	?	+	+	+	+	+	+	+
Liu Y, 2020 [22]	+	?	?	+	+	+	+	+	+	+
Kiyani MM; Butt MA, 2019 [17]	+	?	?	+	+	+	+	+	+	+
Kiyani MM; Sohail MF, 2019 [21]	+	?	?	+	+	+	?	+	+	+
Kiyani MM; Rehman H, 2020 [32]	+	?	?	+	+	+	+	?	+	+
Wang X, 2019 [25]	+	?	?	+	+	?	+	+	+	+
Hao Y, 2019 [24]	+	?	?	+	+	+	?	+	+	+
Zhang J, 2021 [23]	+	?	?	+	+	+	?	+	+	+

+ , indicates low risk of bias; − , indicates high risk of bias; ?, indicates unclear risk of bias

### Meta-analysis and subgroup analysis results

#### Uric acid

**Fig. 2:**
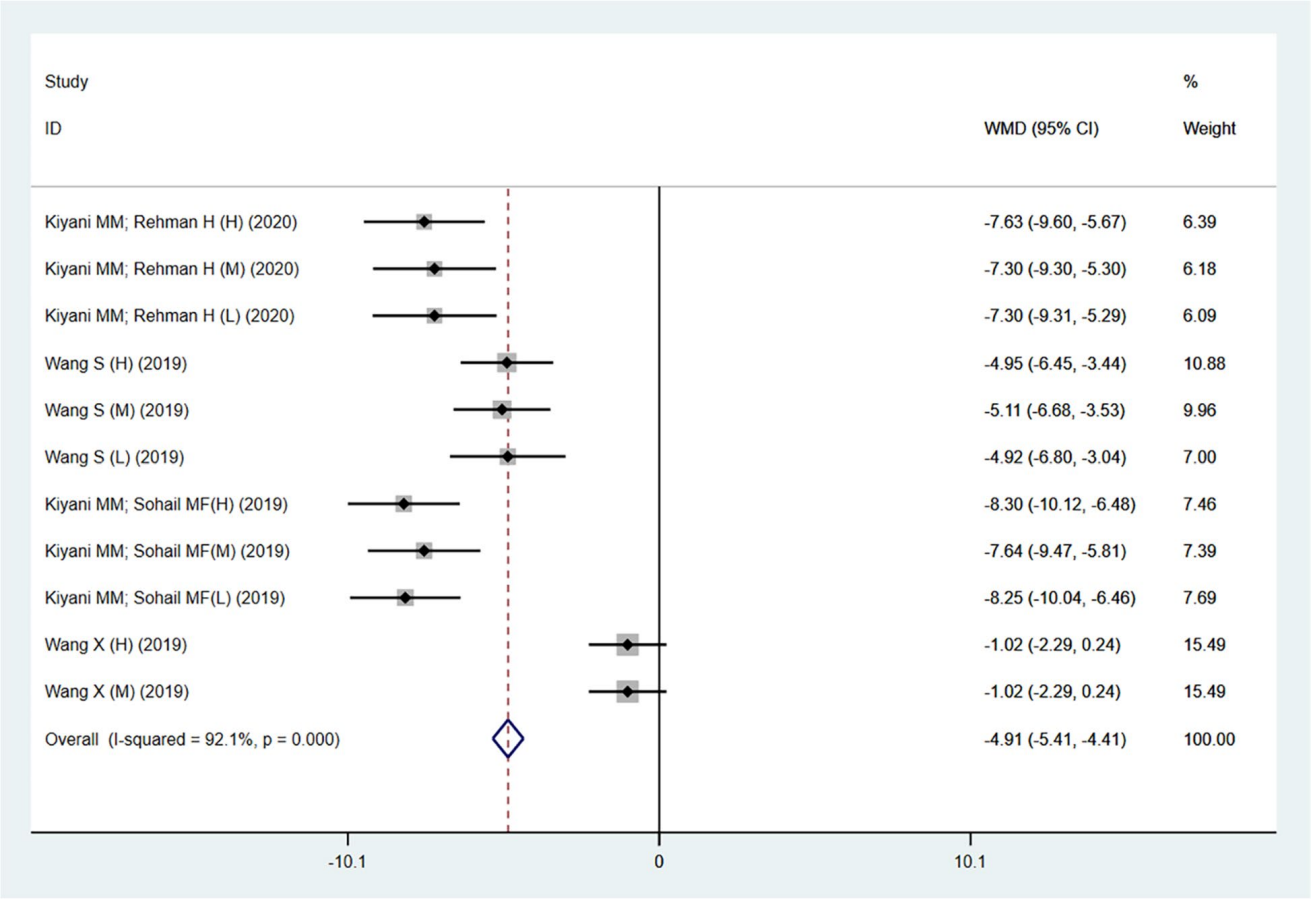
Forest plot of trials investigating the effect of nanoparticles on uric acid levels (compared with the model group). The size of each square represents the weight of each trial, the diamond represents the size of the merger effect. WMD, weighted mean differences; CI, confidence interval. There are four studies including 11 trials reporting the changes in uric acid levels

**Fig. 3:**
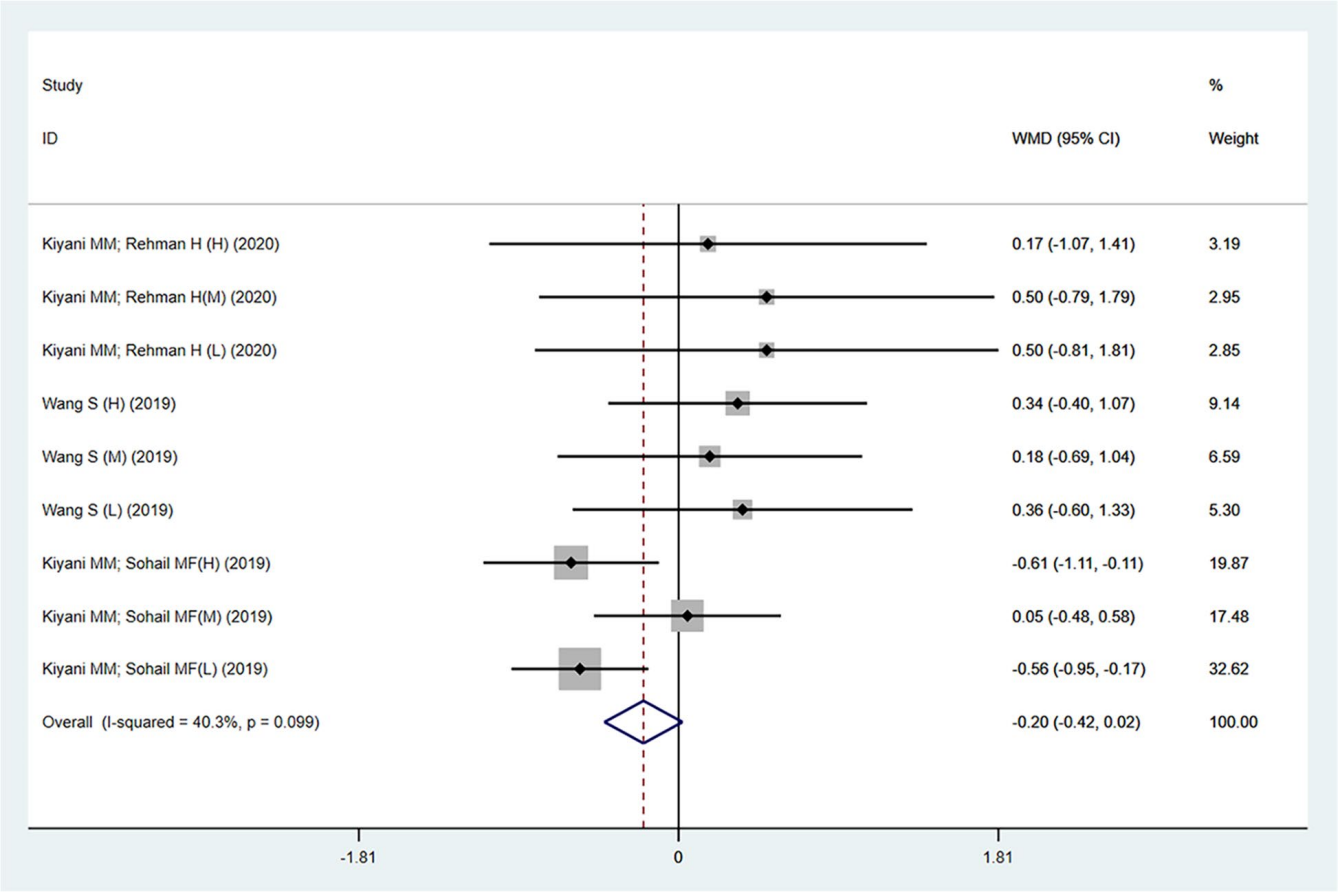
Forest plot of trials investigating the effect of nanoparticles on uric acid levels (compared with allopurinol). The size of each square represents the weight of each trial, the diamond represents the size of the merger effect. WMD, weighted mean differences; CI, confidence interval. There are four studies including 11 trials reporting the changes in uric acid levels

Figure 2 showed the comparison of changes in uric acid levels between nanoparticles and the model group in four studies [20, 21, 25, 32]. The results showed a significant decrease in animal model of gout after nanoparticles treatment (− 4.91; 95%CI: − 5.41 to − 4.41; *p* < 0.001; I^2^ = 92.1%). In addition, the effects of FeO-NPs, nano Ginsenoside Rb1 (nano-GsRb1), ZnO-NPs, IL-1Ra bio-nanoparticles (IK-NPs) and uricase and horseradish peroxidase hybrid CaHPO_4_ nanoflower integrated with a hyaluronic acid dissolvable microneedle system (the uricase& HRP-CaHPO_4_ @HA MN) on the treatment of gouty arthritis were also significant in the remaining six studies (data not shown). Figure 3 showed the comparison of changes in uric acid levels between nanoparticles and allopurinol in three studies [20, 21, 32]. This result revealed that the effect of nanoparticles was no better than allopurinol in reducing uric acid levels, and it also showed substantial heterogeneity (− 0.20; 95%CI: − 0.42 to 0.02; *p* = 0.099; I^2^ = 40.3%). The therapeutic effect of *Puerariae lobatae* Radix carbon dots (PLR-CDs), FeO-NPs, nano-GsRb1, ZnO-NPs, IK-NPs and the uricase& HRP-CaHPO_4_ @HA MN on uric acid levels in animal models of gout were basically similar to that of allopurinol in the remaining seven studies (data was not shown).

The results of subgroup analysis showed that BALB/c mice, six animals, mixed male and female animals, non-metallic or low-dose nanoparticles were significantly more effective than allopurinol in the treatment of mice models with gout induced by MSU crystals. (As shown in Table 3).

**Table 3:** Results of subgroup analysis of included trials in meta-analysis

Groups	n	Allopurinol			n	The model group		
		WMD (95%CI)	*P*-value	I^2^		WMD (95%CI)	*P*-value	I^2^
Material								
Metal	3	0.382(-0.358, 1.121)	0.312	0.00%	3	-7.414(-8.563, -6.264)	0.000	0.00%
Non-metal	6	-0.257(-0.489, -0.024)	0.030	52.90%	8	-4.336(-4.886, -3.785)	0.000	93.30%
Animal species								
BALB/c	6	-0.330(-0.580, -0.081)	0.010	39.50%	6	-7.771(-8.545, -6.997)	0.000	0.00%
SD	3	0.293(-0.191, 0.776)	0.236	0.00%	5	-2.906(-3.553, -2.258)	0.000	88.90%
Dosage								
H	3	-0.265(-0.655, 0.126)	0.185	59.00%	4	-4.486(-5.269, -3.703)	0.001	94.60%
M	3	0.130(-0.297, 0.557)	0.550	0.00%	4	-4.314(-5.109, -3.519)	0.001	93.80%
L	3	-0.366(-0.713, -0.019)	0.039	58.50%	3	-6.850(-7.939, -5.761)	0.000	69.70%
Quantity								
6	6	-0.330(-0.580, -0.081)	0.010	39.50%	8	-4.876(-5.460, -4.291)	0.000	94.50%
8	3	0.293(-0.191, 0.776)	0.236	0.00%	3	-4.998(-5.939, -4.057)	0.000	0.00%
Gender								
F; M	6	-0.330(-0.580, -0.081)	0.010	39.50%	6	-7.771(-8.545, -6.997)	0.000	0.00%
M	3	0.293(-0.191, 0.776)	0.236	0.00%	5	-2.906(-3.553, -2.258)	0.000	88.90%

*F* females, *M* males, *WMD* weighted mean difference, *BALB/c* BALB/c mice, *SD* Sprague Dawley rats, *H* high *M* medium, *L* low

#### The swelling degree of joints

The joint swelling is one of the most prominent symptoms for gouty arthritis. Because the majority of the research described the swelling degree of joints instead of the extractable data, a systematic review was performed in this study on this indicator. As shown in Supplementary Table S3, 13 trials in five studies [16, 20, 22, 23, 25] reported changes in the swelling degree of joints, compared with the model group, five nanoparticles (CuO-NPs, AFIC-CDs, nano-GsRb1, PLR-CDs, and IK-NPs) significantly decreased the diameter of the ankles and effectively relieved ankle swelling in animal models of gout.

#### Nanoparticles safety analysis

##### Blood biochemical index

The results from the blood biochemical tests (Supplementary Table S3) showed that the nanoparticles could significantly reduce the blood urea, creatinine, and uric acid concentrations, and the nanoparticles were more effective than allopurinol in reducing blood urea and creatinine concentrations. Meanwhile, nanoparticles could significantly reduce aspartate aminotransferase (AST), alanine transferase (ALT), and total bilirubin. Furthermore, nanoparticles can reduce cholesterol (TC), low-density lipoprotein (LDL) and triglyceride (TG) concentrations in animal models of gout, and the effect was better than allopurinol. However, the effects of nanoparticles on alkaline phosphatase (ALP) and high-density lipoprotein (HDL) was not significant. To sum up, compared with allopurinol, nanoparticles have basically no damage to the kidney function, liver function and lipid profile of gout animal models. Nanoparticles are safer than allopurinol.

##### Histopathology

The included studies reported that the pathological state after nanoparticle treatment was better than that of the model group and was close to that of the blank group. In rodents with gouty arthritis induced by MSU crystals, hepatocytes were damaged, monocyte cells infiltrated kidney tissue, renal epithelial cells were damaged, and there was considerable inflammatory cell infiltration in joint inflammation. The nanoparticles could relieve renal epithelial cell damage and loss, inflammatory cell infiltration, and joint swelling.

#### Sensitivity analysis and heterogeneity

**Fig. 4:**
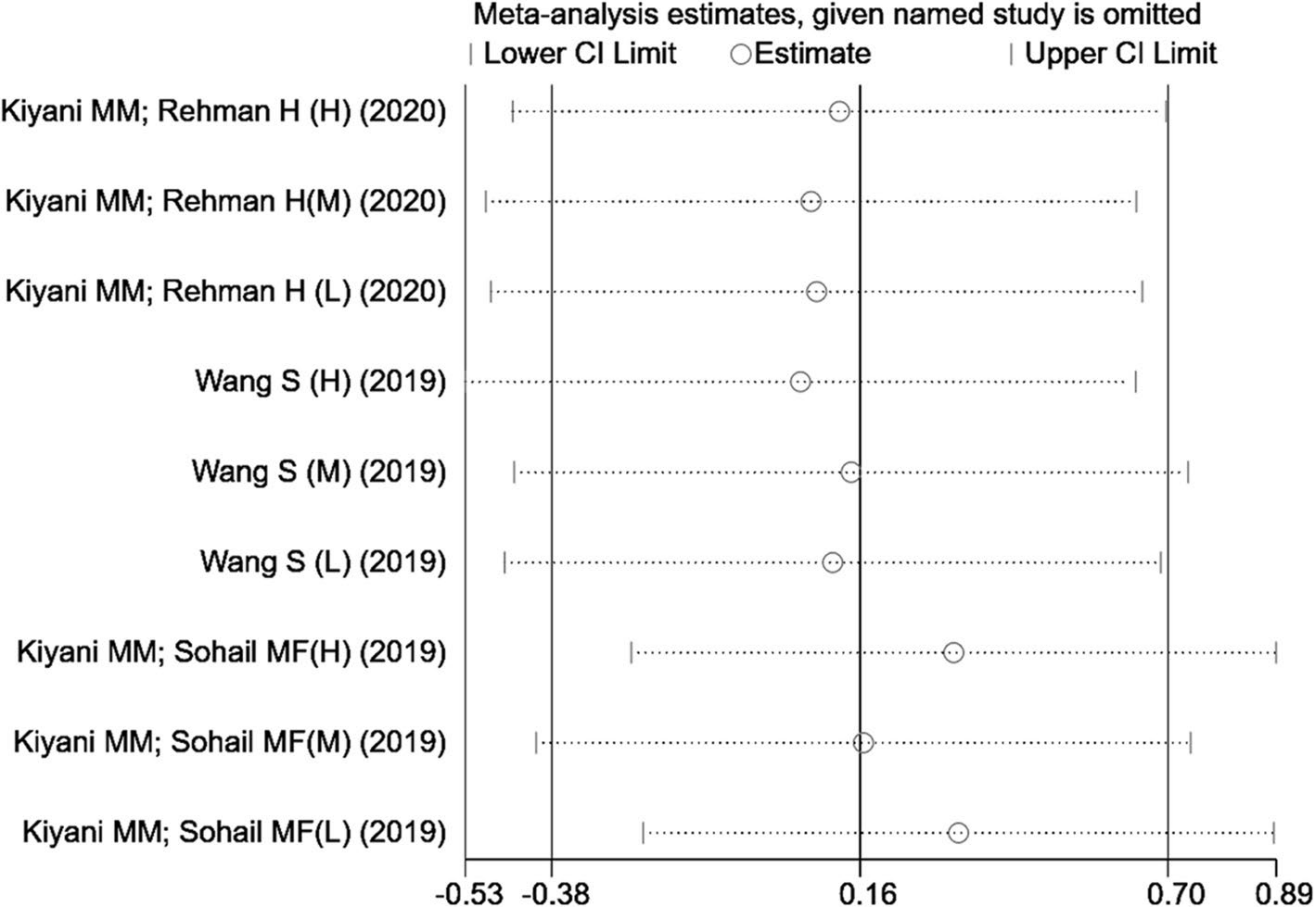
Sensitivity analysis of included studies in uric acid (the model group)

**Fig. 5:**
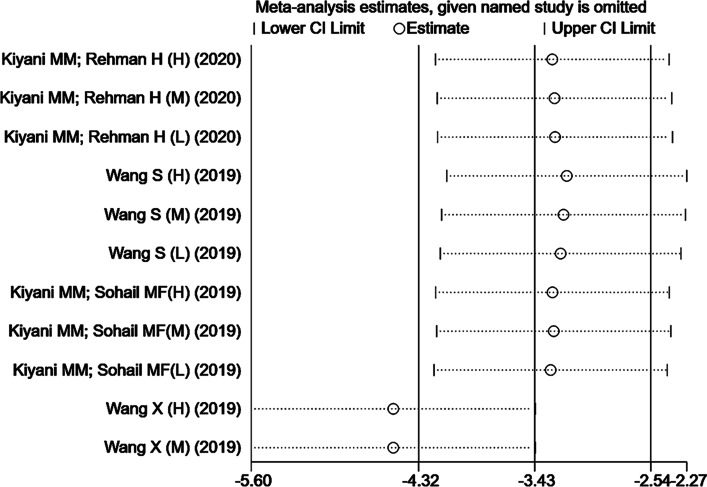
Sensitivity analysis of included studies in uric acid (allopurinol)

This study repeatedly analyzed the impact of a single trial on the overall result by removing a trial in each round. The results of the sensitivity analysis showed that a single trial did not affect the overall significant changes in uric acid levels. When the trial [21] was excluded, the sensitivity analysis results showed that the heterogeneity of uric acid levels was greatly reduced compared with that of the model group (Fig. 4). This indicated that the trial was likely one of the sources of the heterogeneity. Furthermore, when one trial [25] was excluded, the results showed that compared with allopurinol (Fig. 5), the heterogeneity of uric acid levels was reduced, which meant that this study might be one of the sources of high heterogeneity.

Because of the high heterogeneity in the allopurinol (I^2^ = 40.3%) and nanoparticles group (I^2^ = 92.1%), subgroup analyses were performed to explore the possible sources of heterogeneity and showed that nanoparticle encapsulated substance, animal species, nanoparticle dosage, animal quantity, and animal gender were likely the sources of the high heterogeneity in uric acid levels (Table 3).

#### Publication bias

For publication bias, the asymmetry of the funnel plot in Supplementary Figs. S2 and S3 indicated possible publication bias. The results of the Egger’s test indicated a risk of publication bias (*p* = 0.008 compared with allopurinol, and *p* = 0.000 compared with the model group).

## Discussion

This systematic review and meta-analysis aimed to explore the effects of nanoparticles on gouty arthritis in animal models of gout. Overall, the results showed that nanoparticles were effective in reducing uric acid levels, but were not better than allopurinol. It was mentioned that the nanoparticles were safer than allopurinol, and had less damage to kidney function, liver function, and lipid profile. Meanwhile, nanoparticles could reduce the degree of joint swelling and ankle diameter. Although the results are still uncertain, nanoparticles may be a safe and effective intervention for gouty arthritis, and nanoparticles may be used for preclinical medication of gouty arthritis in the future.

The use of nanoparticles in the treatment of gouty arthritis has several advantages. Firstly, nanoparticles could effectively reduce the serum uric acid level thus treatment of gouty arthritis. This mainly relied on the pharmacological properties of the encapsulated substances in nanoparticles and the effective delivery of nanomaterial shells. Several nanoparticle encapsulated substances in this study had the characteristics of anti-inflammation, control of oxidative stress, and analgesia. However, poor water solubility, low bioavailability, and short half-life limited their therapeutic effects. Therefore, the nanomaterial shell was used as an effective delivery tool, which could prolong the biological half-life, improve the pharmacokinetics, and maximize the therapeutic effect of the substances [35]. Secondly, nanoparticles could effectively improve the bioavailability of medications. In the study of nano-GsRb1 [22], the bioavailability of the substance was increased when the substance was encapsulated in nanomaterials to form nanoparticles. Win et al. [36] also found that Vitamin E succinated polyethylene glycol 1000-emulsified Poly (D, L-lactic-co-glycolic acid) nanoparticles was used in the paclitaxel formulation to improve their therapeutic index. This was mainly caused by the properties of the nanoparticle entrapment of substances, which reduced their loss before reaching the designated site of action. What’s more, nanoparticles also increased their bioavailability to enable the target organ to utilize the substances to the maximum extent. In addition, their negative effects were reduced, which in turn promoted the therapeutic effect of the substances on gouty arthritis. Thirdly, nanoparticles were safer than allopurinol. The results of this study showed that nanoparticles had a high level of safety for the kidney and liver. The safety of substances was mainly reflected in their toxicity to non-target organs [37]. After nanoparticle entrapment of substances, the substances could be delivered to the target location, reducing the side effects of the substances off-target, and protecting other organs and tissues more safely and effectively [38–40]. In addition, several nanoparticles [32, 33] were shown to cause liver inflammation, which was mainly related to liver dysfunction or bile duct blockage. However, further discussion on this issue is warranted in the future. Lastly, the results also showed that nanoparticles could effectively reduce the toxicity and side effects of substances [15]. The studies on quartz [41, 42] had similar results. For example, the toxicity of toxic quartz diminished after being wrapped with a polyvinylpyridine-N-oxide-polymer, and the impact of toxic quartz on cells was reduced. Moreover, the nanoparticle entrapment of paclitaxel reduced paclitaxel toxicity [36]. The results of the included study [32] showed that CuSO_4_ could only slightly reduce the uric acid level in gout animal models and CuSO_4_ had a damaging effect on liver function, after the nanoparticles entrapment of copper oxide, CuO-NPs could significantly reduce the uric acid level and the damage to kidney and liver function could be reduced.

The results also showed that the effect of treating gout might be related to the species, gender, and quantity of animals used in the animal experiments and the dose, material of the nanoparticle entrapment of medications. The subgroup analysis of animal species revealed that the experimental effects of using BALB/c mice were greater compared with those of using SD rats. Currently, gout model animals include rats [43], mice [44, 45], rabbits [46], chickens [47], and zebrafish [48]. Studies have shown that the model established by MSU injection into the left ankle joint of chickens is an ideal model for studying gouty arthritis [47]. In the future, it needs to further explore suitable animals for gouty arthritis model. In addition, the subgroup analysis of animal gender showed that the gout treatment effect of using mixed males and females was greater than that of using only males. The results of one study [49] reported that the incidence of gouty arthritis in men was higher than that in women, and women were more likely to suffer from gouty arthritis after menopause [50]. Thus, the occurrence of gouty arthritis might be related to decreased estrogen levels [51]. In postmenopausal women, more attention should be paid to the prevention of gouty arthritis. Moreover, the subgroup analysis showed that in reducing the serum uric acid level, low-dose nanoparticle-encapsulated medications were more effective than high-dose and medium-dose nanoparticle-encapsulated medications. The effect of nanoparticle-encapsulated medications achieves saturation when a certain dose is reached. Excessive medications will accumulate in other non-target organs such as the renal and liver, and result in side effects. It has been found that the rate of nanoparticle-encapsulated medications association decreases with the number of associated nanoparticle-encapsulated medications rising, becoming saturated [52]. Meanwhile, Tian et al. administered the nanoscale vanadium dioxide particle (SVO_2_) to mice by gavage, and they found that a higher dosage could lead to higher vanadium contents in organs and accumulation in bones and liver [53]. Therefore, the appropriate dosage of medication can maximize effectiveness while minimizing harm to the body. Researchers should conduct further studies to explore the optimal medication dose. Furthermore, sources of heterogeneity also included animal quantity. The number of animals used an experiment is related to the applicability and persuasiveness of the experiment. Appropriate animal numbers for experiments should be fully explored. Finally, the results showed that nanoparticle encapsulated substance was an important consideration for the effectiveness of gouty arthritis treatment. The subgroup analysis showed that compared with the gout model group, the effect of metallic nanoparticles on reducing the serum uric acid level was better than that of non-metallic nanoparticles. Cu, Fe, and Zn were essential trace elements for the human body. Compared with non-metallic herbal medicines (such as turmeric, GsRb1, PLR, AFI, etc.), they had better water solubility and higher bioavailability [20–22, 25]. After being encapsulated by nanomaterials, they could be quickly absorbed and utilized, and had good anti-oxidation and anti-gout effects [17, 32, 33]. However, compared with non-metallic nanoparticles, metallic nanoparticles were less safe since they were more toxic to the liver, spleen, kidney, and other organs than non-metallic nanoparticles [16, 17, 32, 33]. Therefore, more studies will be needed to explore the optimal substance species encapsulated in nanoparticles in the future.

The main advantage of this systematic review was that we assessed the therapeutic effects of nanoparticles in animal models of gout. The results of this meta-analysis would provide a reference for the further development of nanomedicine. However, the present study still had some certain limitations. Firstly, the number of included studies and sample sizes were insufficient. Secondly, the included studies in the present meta-analysis were only published in English, which could be incomprehensive. Furthermore, the heterogeneity of the meta-analysis was high, but this was unavoidable because the studies we included varied in species, quantity, and gender of animals, dosage, and nanoparticle encapsulated substances. Therefore, subgroup analyses and sensitivity analyses were used to explore possible sources of heterogeneity. Finally, the funnel plots of nanoparticles compared with the model group and allopurinol were asymmetric, and Egger’s test also suggested the possibility of publication bias. This might be related to the selective reporting of studies, with only four of the studies included in the review being able to extract data for meta-analysis. Meanwhile, to a certain extent, it showed that the research in this area was still in the development stage and had great application prospects. In the future, more high-quality literature would be needed to support the research ideas.

## Conclusion

This systematic review and meta-analysis revealed that nanoparticles could effectively reduce the level of uric acid in animal models of gout. Nanoparticles might become effective medications in the treatment of gouty arthritis because of its safety and efficacy, but the results are inconclusive. In the future, larger sample size, longer duration, and well-designed trials are required to demonstrate that nanoparticles can be used in preclinical treatment of gouty arthritis.

## Supplementary Information

**Additional file 1: Supplementary Table S1. ** PRISMA 2020 Checklist.

**Additional file 2: Supplementary Table S2. **Search strategy used in PubMed/ Scopus/ Web of Science / the Cochrane library/ Embase online database. **Supplementary Table S3. **Important results on the swelling degree of joints and blood biochemical index from studies without meta-analyzed. **Supplementary Fig. S1. **Quality assessment of included studies using SYRCLE’s risk of bias tool. **Supplementary Figure S2. **Funnel plot for the association between nanoparticles and allopurinol. **Supplementary Figure S3. ** Funnel plot for the association between nanoparticles and the model group.

## Data Availability

All data generated or analyzed during this study are included in this published article [and its supplementary information files].

## References

[1] Dalbeth N, Merriman TR, Stamp LK (2016). Gout. Lancet.

[2] Ichida K, Matsuo H, Takada T, Nakayama A, Murakami K, Shimizu T, Yamanashi Y, Kasuga H, Nakashima H, Nakamura T (2012). Decreased extra-renal urate excretion is a common cause of hyperuricemia. Nat Commun.

[3] Nuki G, Simkin PA (2006). A concise history of gout and hyperuricemia and their treatment. Arthritis Res Ther.

[4] Dehlin M, Jacobsson L, Roddy E (2020). Global epidemiology of gout: prevalence, incidence, treatment patterns and risk factors. Nat Rev Rheumatol.

[5] FitzGerald JD, Dalbeth N, Mikuls T, Brignardello-Petersen R, Guyatt G, Abeles AM, Gelber AC, Harrold LR, Khanna D, King C (2020). 2020 American college of rheumatology guideline for the management of gout. Arthritis Care Res (Hoboken).

[6] Wilson L, Saseen JJ (2016). gouty arthritis: a review of acute management and prevention. Pharmacotherapy.

[7] Lee JW, Lee KH (2019). Comparison of renoprotective effects of febuxostat and allopurinol in hyperuricemic patients with chronic kidney disease. Int Urol Nephrol.

[8] Chohan S (2011). Safety and efficacy of febuxostat treatment in subjects with gout and severe allopurinol adverse reactions. J Rheumatol.

[9] Liang G, Nie Y, Chang Y, Zeng S, Liang C, Zheng X, Xiao D, Zhan S, Zheng Q (2019). Protective effects of Rhizoma smilacis glabrae extracts on potassium oxonate- and monosodium urate-induced hyperuricemia and gout in mice. Phytomedicine.

[10] Wagner AJ, Ravi V, Riedel RF, Ganjoo K, Van Tine BA, Chugh R, Cranmer L, Gordon EM, Hornick JL, Du H (2021). nab-Sirolimus for patients with malignant perivascular epithelioid cell tumors. J Clin Oncol.

[11] Lawitz EJ, Shevell DE, Tirucherai GS, Du S, Chen W, Kavita U, Coste A, Poordad F, Karsdal M, Nielsen M (2022). BMS-986263 in patients with advanced hepatic fibrosis: 36-week results from a randomized, placebo-controlled phase 2 trial. Hepatology.

[12] Lautenschläger C, Schmidt C, Lehr CM, Fischer D, Stallmach A (2013). PEG-functionalized microparticles selectively target inflamed mucosa in inflammatory bowel disease. Eur J Pharm Biopharm.

[13] Ahmadi M, Hajialilo M, Dolati S, Eghbal-Fard S, Heydarlou H, Ghaebi M, Ghassembaglou A, Aghebati-Maleki L, Samadi Kafil H, Kamrani A (2020). The effects of nanocurcumin on Treg cell responses and treatment of ankylosing spondylitis patients: A randomized, double-blind, placebo-controlled clinical trial. J Cell Biochem.

[14] Najahi-Missaoui W, Arnold RD, Cummings BS. Safe Nanoparticles: Are We There Yet? Int J Mol Sci. 2020;22(1):385. 10.3390/ijms22010385 PMC7794803 33396561

[15] De Jong WH, Borm PJ (2008). Drug delivery and nanoparticles:applications and hazards. Int J Nanomedicine.

[16] Kiyani MM, Butt MA, Rehman H, Mustafa M, Sajjad AG, Shah SSH, Mahmood T, Bokhari SAI. Evaluation of antioxidant activity and histopathological changes occurred by the oral ingestion of CuO nanoparticles in monosodium urate crystal-induced hyperuricemic BALB/c mice. Biol Trace Elem Res. 2022;200(1):217–27. 10.1007/s12011-021-02615-3 33594526

[17] Kiyani MM, Butt MA, Rehman H, Ali H, Hussain SA, Obaid S, Arif Hussain M, Mahmood T, Bokhari SAI (2019). Antioxidant and anti-gout effects of orally administered zinc oxide nanoparticles in gouty mice. J Trace Elem Med Biol.

[18] Chen Y, Zhang Q, Qin X, Li J, Zhao Y, Xia Y (2022). superparamagnetic iron oxide nanoparticles protect human gingival fibroblasts from porphyromonas gingivalis invasion and inflammatory stimulation. Int J Nanomedicine.

[19] Xie H, Chen Y, Du K, Wu W, Feng X (2020). Puerarin alleviates vincristine-induced neuropathic pain and neuroinflammation via inhibition of nuclear factor-κB and activation of the TGF-β/Smad pathway in rats. Int Immunopharmacol.

[20] Wang S, Zhang Y, Kong H, Zhang M, Cheng J, Wang X, Lu F, Qu H, Zhao Y (2019). Antihyperuricemic and anti-gouty arthritis activities of Aurantii fructus immaturus carbonisata-derived carbon dots. Nanomedicine.

[21] Kiyani MM, Sohail MF, Shahnaz G, Rehman H, Akhtar MF, Nawaz I, Mahmood T, Manzoor M, Bokhari SAI (2019). Evaluation of turmeric nanoparticles as anti-gout agent: modernization of a traditional drug. Medicina Kaunas.

[22] Liu Y, Zhu H, Zhou W, Ye Q (2020). Anti-inflammatory and anti-gouty-arthritic effect of free Ginsenoside Rb1 and nano Ginsenoside Rb1 against MSU induced gouty arthritis in experimental animals. Chem Biol Interact.

[23] Zhang J, Sun Y, Qu Q, Li B, Zhang L, Gu R, Zuo J, Wei W, Ma C, Liu L (2021). Engineering non-covalently assembled protein nanoparticles for long-acting gouty arthritis therapy. J Mater Chem B.

[24] Hao Y, Li H, Cao Y, Chen Y, Lei M, Zhang T, Xiao Y, Chu B, Qian Z (2019). Uricase and horseradish peroxidase hybrid CaHPO4 nanoflower integrated with transcutaneous patches for treatment of hyperuricemia. J Biomed Nanotechnol.

[25] Wang X, Zhang Y, Zhang M, Kong H, Wang S, Cheng J, Qu H, Zhao Y (2019). Novel carbon dots derived from puerariae lobatae radix and their anti-gout effects. Molecules.

[26] Cumpston M, Li T, Page MJ, Chandler J, Welch VA, Higgins JP, Thomas J (2019). Updated guidance for trusted systematic reviews: a new edition of the cochrane handbook for systematic reviews of interventions. Cochrane Database Syst Rev.

[27] Moher D, Liberati A, Tetzlaff J, Altman DG, Group P (2009). Preferred reporting items for systematic reviews and meta-analyses: the PRISMA statement. PLoS Med.

[28] Hooijmans CR, Rovers MM, de Vries RB, Leenaars M, Ritskes-Hoitinga M, Langendam MW. SYRCLE’s risk of bias tool for animal studies. BMC Med Res Methodol. 2014;14:43. 10.1186/1471-2288-14-43 PMC4230647 24667063

[29] Chalmers I (1993). The Cochrane collaboration: preparing, maintaining, and disseminating systematic reviews of the effects of health care. Ann N Y Acad Sci.

[30] Tufanaru C, Munn Z, Stephenson M, Aromataris E (2015). Fixed or random effects meta-analysis? Common methodological issues in systematic reviews of effectiveness. Int J Evid Based Healthc.

[31] Sterne JA, Gavaghan D, Egger M (2000). Publication and related bias in meta-analysis: power of statistical tests and prevalence in the literature. J Clin Epidemiol.

[32] Kiyani MM, Rehman H, Hussain MA, Jahan S, Afzal M, Nawaz I, Mahmood T, Bokhari SAI (2020). Inhibition of Hyperuricemia and Gouty Arthritis in BALB/c Mice Using Copper Oxide Nanoparticles. Biol Trace Elem Res.

[33] Kiyani MM, Moghul NB, Butt MA, Rehman H, Masood R, Rajput TA, Bokhari SAI. Anti-hyperuricemic effect of iron oxide nanoparticles against monosodium urate crystals induced gouty arthritis in BALB/c mice. Biol Trace Elem Res. 2022;200(4):1659–66. 10.1007/s12011-021-02769-0 34196880

[34] Nistor M, Behringer W, Schmidt M, Schiffner R (2017). A systematic review of neuroprotective strategies during hypovolemia and hemorrhagic shock. Int J Mol Sci.

[35] Kim TH, Jiang HH, Youn YS, Park CW, Lim SM, Jin CH, Tak KK, Lee HS, Lee KC (2011). Preparation and characterization of Apo2L/TNF-related apoptosis-inducing ligand-loaded human serum albumin nanoparticles with improved stability and tumor distribution. J Pharm Sci.

[36] Win KY, Feng SS (2006). In vitro and in vivo studies on vitamin E TPGS-emulsified poly(D, L-lactic-co-glycolic acid) nanoparticles for paclitaxel formulation. Biomaterials.

[37] Riley RS, June CH, Langer R, Mitchell MJ (2019). Delivery technologies for cancer immunotherapy. Nat Rev Drug Discov.

[38] Shao K, Singha S, Clemente-Casares X, Tsai S, Yang Y, Santamaria P (2015). Nanoparticle-based immunotherapy for cancer. ACS Nano.

[39] Toy R, Roy K (2016). Engineering nanoparticles to overcome barriers to immunotherapy. Bioeng Transl Med.

[40] Moon JJ, Huang B, Irvine DJ (2012). Engineering nano- and microparticles to tune immunity. Adv Mater.

[41] Albrecht C, Knaapen AM, Becker A, Höhr D, Haberzettl P, van Schooten FJ, Borm PJ, Schins RP (2005). The crucial role of particle surface reactivity in respirable quartz-induced reactive oxygen/nitrogen species formation and APE/Ref-1 induction in rat lung. Respir Res.

[42] Schins RP, Duffin R, Höhr D, Knaapen AM, Shi T, Weishaupt C, Stone V, Donaldson K, Borm PJ (2002). Surface modification of quartz inhibits toxicity, particle uptake, and oxidative DNA damage in human lung epithelial cells. Chem Res Toxicol.

[43] Zhou M, Ze K, Hua L, Liu L, Kuai L, Zhang M, Li B, Wang Y, Li X (2020). Cyr61 Promotes Inflammation of a Gouty Arthritis Model in Rats. Mediators Inflamm.

[44] Lee YM, Cho SN, Son E, Song CH, Kim DS (2020). Apamin from bee venom suppresses inflammation in a murine model of gouty arthritis. J Ethnopharmacol.

[45] Caution K, Young N, Robledo-Avila F, Krause K, Abu Khweek A, Hamilton K, Badr A, Vaidya A, Daily K, Gosu H (2019). Caspase-11 mediates neutrophil chemotaxis and extracellular trap formation during acute gouty arthritis through alteration of cofilin phosphorylation. Front Immunol.

[46] Hu Y, Yang Q, Gao Y, Guo X, Liu Y, Li C, Du Y, Gao L, Sun D, Zhu C (2019). Better understanding of acute gouty attack using CT perfusion in a rabbit model. Eur Radiol.

[47] Liu RH, Shi W, Zhang YX, Zhuo M, Li XH. Selective inhibition of adenylyl cyclase subtype 1 reduces inflammatory pain in chicken of gouty arthritis. Mol Pain. 2021;17:17448069211047864. 10.1177/17448069211047863 PMC8591642 34761717

[48] Hall CJ, Sanderson LE, Lawrence LM, Pool B, van der Kroef M, Ashimbayeva E, Britto D, Harper JL, Lieschke GJ, Astin JW (2018). Blocking fatty acid-fueled mROS production within macrophages alleviates acute gouty inflammation. J Clin Invest.

[49] Zhu Y, Pandya BJ, Choi HK (2011). Prevalence of gout and hyperuricemia in the US general population: the national health and nutrition examination survey 2007–2008. Arthritis Rheum.

[50] Hak AE, Curhan GC, Grodstein F, Choi HK (2010). Menopause, postmenopausal hormone use and risk of incident gout. Ann Rheum Dis.

[51] Marinello E, Riario-Sforza G, Marcolongo R (1985). Plasma follicle-stimulating hormone, luteinizing hormone, and sex hormones in patients with gout. Arthritis Rheum.

[52] Faria M, Noi KF, Dai Q, Björnmalm M, Johnston ST, Kempe K, Caruso F, Crampin EJ (2019). Revisiting cell-particle association in vitro: a quantitative method to compare particle performance. J Control Release.

[53] Tan SY, Chen XZ, Cao A, Wang H. Biodistribution of vanadium dioxide particles in mice by consecutive gavage administration: effects of particle size, dosage, and health condition of mice. Biol Trace Elem Res. 2022. 10.1007/s12011-022-03395-0. 10.1007/s12011-022-03395-0 35984600

