# Supplementary material for: Effect of nanoparticles on gouty arthritis: a systematic review and meta-analysis
Source: BMC Musculoskelet Disord. 2023 Feb 14;24:124. doi: 10.1186/s12891-023-06186-3 (PMC9926759; doi:10.1186/s12891-023-06186-3)

***SUPPLEMENTARY INFORMATION***

**Legends**

**Supplementary Table S1** PRISMA 2020 Checklist.

**Supplementary Table S2** Search strategy used in PubMed/ Scopus/ Web of Science/ the Cochrane library/ Embase online database.

**Supplementary Table S3** Important results on the swelling degree of joints and blood biochemical index from studies without meta-analyzed.

**Supplementary Fig. S1** Quality assessment of included studies using SYRCLE’s risk of bias tool.

**Supplementary Fig. S2** Funnel plot for the association between nanoparticles and allopurinol.

**Supplementary Fig. S3** Funnel plot for the association between nanoparticles and the model group.

**Supplementary Table S2.** Search strategy used in PubMed/ Scopus/ Web of Science / Cochran library/ Embase online database.

| Database name | Search strategies: key words and how these were combined in the search | Laster update | Number of studies identified |
| --- | --- | --- | --- |
| PubMed | (("Arthritis, Gouty"[Mesh]) OR ((((((((Gouty Arthritis[Title/Abstract]) OR (Arthritides, Gouty[Title/Abstract])) OR (Gouty Arthritides[Title/Abstract])) OR (Synovial joints[Title/Abstract])) OR (Gout[Title/Abstract])) OR (Uric acid[Title/Abstract])) OR (hyperuricemi*[Title/Abstract])) OR (uric acid crystals[Title/Abstract]))) AND (("Nanostructures"[Mesh]) OR (((((((Nanostructure[Title/Abstract]) OR (Nanostructured Materials[Title/Abstract])) OR (Material, Nanostructured[Title/Abstract])) OR (Materials, Nanostructured[Title/Abstract])) OR (Nanostructured Material[Title/Abstract])) OR (Nanomaterials[Title/Abstract])) OR (Nanomaterial[Title/Abstract]))) | 21^th^  April, 2022 | 845 |
| Scopus | ((TITLE-ABS-KEY (arthritis, AND gouty) OR TITLE-ABS-KEY (gouty AND arthritis) OR TITLE-ABS-KEY (arthritides, AND gout) OR TITLE-ABS-KEY (gouty AND arthritides) OR TITLE-ABS-KEY (synovial AND joints) OR TITLE-ABS-KEY (gout) OR TITLE-ABS-KEY (uric AND acid) OR TITLE-ABS-KEY (hyperuricemi*) OR TITLE-ABS-KEY (uric AND acid AND crystals))) AND ((TITLE-ABS-KEY (nanostructures) OR TITLE-ABS-KEY (nanostructure) OR TITLE-ABS-KEY (nanostructured AND materials) OR TITLE-ABS-KEY (material, AND nanostructured) OR TITLE-ABS-KEY (materials, AND nanostructured) OR TITLE-ABS-KEY (nanostructured AND material) OR TITLE-ABS-KEY (nanomaterials ) OR TITLE-ABS-KEY (nanomaterial))) | 21^th^  April, 2022 | 716 |
| Web of science | #1 TOPIC: (Arthritis, Gouty) OR TOPIC: (Gouty Arthritis) OR TOPIC: (Arthritides, Gouty) OR TOPIC: (Gouty Arthritides) OR TOPIC: (Synovial joints) OR TOPIC: (Gout) OR TOPIC: (Uric acid) OR TOPIC: (hyperuricemi*) OR TOPIC: (uric acid crystals) 139789  #2 TOPIC: (Nanostructures) OR TOPIC: (Nanostructure) OR TOPIC: (Nanostructured Materials) OR TOPIC: (Material, Nanostructured) OR TOPIC: (Materials, Nanostructured) OR TOPIC: (Nanostructured Material) OR TOPIC: (Nanomaterials) OR TOPIC: (Nanomaterial) 749252  #3 #1AND#2 1683 | 21^th^  April, 2022 | 1683 |
| Cochran library | # 1 MeSH descriptor: [Arthritis, Gouty] explode all trees 62  # 2 (Gouty Arthritis):ti,ab,kw OR (Arthritides, Gouty):ti,ab,kw OR (Gouty Arthritides):ti,ab,kw OR (Synovial joints):ti,ab,kw OR (Gout):ti,ab,kw (Word variations have been searched) 2107  # 3 (Uric acid):ti,ab,kw OR (hyperuricemi*):ti,ab,kw OR (uric acid crystals):ti,ab,kw (Word variations have been searched) 5140  # 4 #1 OR #2 OR #3 6272  # 5 MeSH descriptor: [Nanostructures] explode all trees 387  # 6 (Nanostructure):ti,ab,kw OR (Nanostructured Materials):ti,ab,kw OR (Material, Nanostructured):ti,ab,kw OR (Materials, Nanostructured):ti,ab,kw OR (Nanostructured Material):ti,ab,kw (Word variations have been searched) 35  # 7 (Nanomaterials):ti,ab,kw OR (Nanomaterial):ti,ab,kw (Word variations have been searched) 24  # 8 #5 OR #6 OR #7 425  # 9 #4 AND #8 0 | 21^th^  April, 2022 | 0 |
| Embase | #1 ‘gout’/exp OR ‘arthritis, gouty’:ti,ab,kw OR ‘gouty arthritis’:ti,ab,kw OR ‘arthritides, gouty’:ti,ab,kw OR ‘gouty arthritides’:ti,ab,kw OR ‘synovial joints’:ti,ab,kw OR ‘uric acid’:ti,ab,kw OR ‘hyperuricemi’:ti,ab,kw OR ‘uric acid crystals’:ti,ab,kw 69554  #2 ‘nanomaterial’/exp OR ‘nanostructures’:ti,ab,kw OR ‘nanostructure’:ti,ab,kw OR ‘nanostructured materials’:ti,ab,kw OR ‘material, nanostructured’:ti,ab,kw OR ‘materials, nanostructured’:ti,ab,kw OR ‘nanostructured material’:ti,ab,kw OR ‘nanomaterials’:ti,ab,kw 4364415  #3 #1 AND #2 1163 | 21^th^  April, 2022 | 1163 |

**Supplementary Table S3** Important results on the swelling degree of joints and blood biochemical index from studies without meta-analyzed.

| Study | Intervention | The swelling degree of joints | Blood biochemical index | | |
| --- | --- | --- | --- | --- | --- |
|  |  |  | kidney function | Liver function | Lipid profile |
| Kiyani MM; Butt MA | CuO-NPs | NA | NA | NA | NA |
| Wang S | AFIC-CDs | Compared with model group, the paw volume and the diameter of the ankles of the rats were reduced considerably in rats treated with AFIC-CDs at doses of 2, 4 and 8 mg/kg at 12 and 24 h, and the paw pressure scores in the 4 and 8 mg/kg AFIC-CDs groups reduced at 12 h and 24 h after MSU injection. | NA | NA | NA |
| Kiyani MM; Moghul NB | FeO-NPs | NA | FeO-NP groups showed decreasing levels of creatinine (P <0.005) and blood urea (P <0.005). | Compared with the model group, the ALT, AST, ALP and total bilirubin of only 5ppm FeO-NPs group decreased (P <0.005). | Lipid profile found decreased cholesterol and LDL levels in groups treated with 10 and 20 ppm FeO-NPs as compared to other treated groups (P <0.005). Groups treated with 50 mg/kg and 100 mg/kg allopurinol were found to have raised triglycerides values (P <0.005). |
| Liu Y | GsRb1;  nano-GsRb1 | Nano-GsRb1 was responsible for the inhibition of edema at 4 h, 8 h, 12 h, 24 h, 48 h post injecting monosodium rate. Pretreatment with nano-GsRb1 significantly prevented the ankle swelling as compared to MSU crystals GsRb1. Significant amelioration in both paw swelling and bone destruction along with reduction in radiological bone destruction score was seen in response to treatment with GsRb1 and nano-GsRb1. | NA | NA | NA |
| Kiyani MM; Butt MA | ZnO-NPs | NA | The MSU crystal-induced mice had a significant increase (P < 0.001) in urea and creatinine levels while, in ZnO-NPs (P < 0.001) and allopurinol (P < 0.05) treated mice had a significant decrease in urea level as compared to control | ZnO-NPs (5,10, and 20 ppm) treated group showed significant decrease in ALT (P < 0.05) and AST (P < 0.01) values. Evaluation of total bilirubin revealed that its values increase significantly in MSU treated group, while other groups did not show any significant change. | All treated groups showed significant (P <0.001) decrease in TC, LDL, and TG in comparison with control. The HDL level significantly (P <0.001) decreased in MSU treated group while, it showed significant (P < 0.01), (P < 0.05) increase in ZnO-NPs (20 ppm) and allopurinol treated groups respectively |
| Kiyani MM; Sohail MF | T-NPs | NA | All treatments decreased the level of urea, creatinine and uric acid as compared to diseased control group, indicating the success of the therapy. | Compared with the model group, the ALT, AST and ALP of the 5,20 ppmT-NPs groups were all lower, and the effect was better than that of allopurinol, with statistical significance. Total bilirubin showed no significant deviation in any group as compared with control, showing all values within the acceptable ranges. | T-NPs significantly (p <0.05) lowered the TC, TG and LDL levels in arthritic mice at 10 and 20 ppm, and the effect was better than that of allopurinol. |

**Continued Supplementary Table S3**

| Study | Intervention | The swelling degree of joints | Blood biochemical index | | |
| --- | --- | --- | --- | --- | --- |
|  |  |  | kidney function | Liver function | Lipid profile |
| Kiyani MM; Rehman H | CuO-NPs | It was observed that the mice treated with CuO nanoparticles showed a decrease in synovial joints inflammation in comparison with negative control. | Groups treated with CuO NPs also showed reduced creatinine (P <0.005) and blood urea levels (P <0.005). | The ALT of 5, 10ppm CuO-NPs groups, and the AST, ALP, and bilirubin of 5, 10, 20ppm CuO-NPs groups were lower than those of the model group (P <0.005) and allopurinol group. | Compared with the model group, the TC, LDL and HDL of 5,10,20 ppm CuO-NPS group decreased, and the TG of 10ppmCuO-NPs group decreased (P <0.005), and the effect was better than that of allopurinol. |
| Wang X | PLR-CDs | PLR-CDs minimized the ankle swelling from 1 h to 24 h, showing a similar effect to that of the positive drug. | NA | NA | NA |
| Hao Y | The uricase& HRP-CaHPO4 @HA MN | NA | NA | NA | NA |
| Zhang J | IK-NPs | The IK-Np-treated rats exhibited significant and sustained detumescence effect after the 24th hour than the indomethacin group. Moreover, the effect of IK-Pr nanoassembly stimulated an extended-retention time after the 48th hour, and lasted to the 72nd hour. | NA | NA | NA |
| CuO-NPs, copper oxide nanoparticles; AFIC-CDs, Aurantii fructus immaturus carbonisata-derived carbon dots; FeO-NPs, iron oxide nanoparticles; GsRb1, Ginsenoside Rb1; ZnO-NPs, zinc oxide nanoparticles; T-NPs, turmeric nanoparticles; PLR-CDs, Puerariae lobatae Radix carbon dots; IK-NPs, IL-1Ra bio-nanoparticles; AST, aspartate aminotransferase; ALT, alanine transferase; ALP, alkaline phosphatase; TC, cholesterol; LDL, low-density lipoprotein; HDL, high-density lipoprotein; TG, triglyceride; h, hour; NA, not applicable. | | | | | |

**Supplementary Fig. S1** Quality assessment of included studies using SYRCLE’s risk of bias tool.

**Supplementary Figure S2.** Funnel plot for the association between nanoparticles and allopurinol.


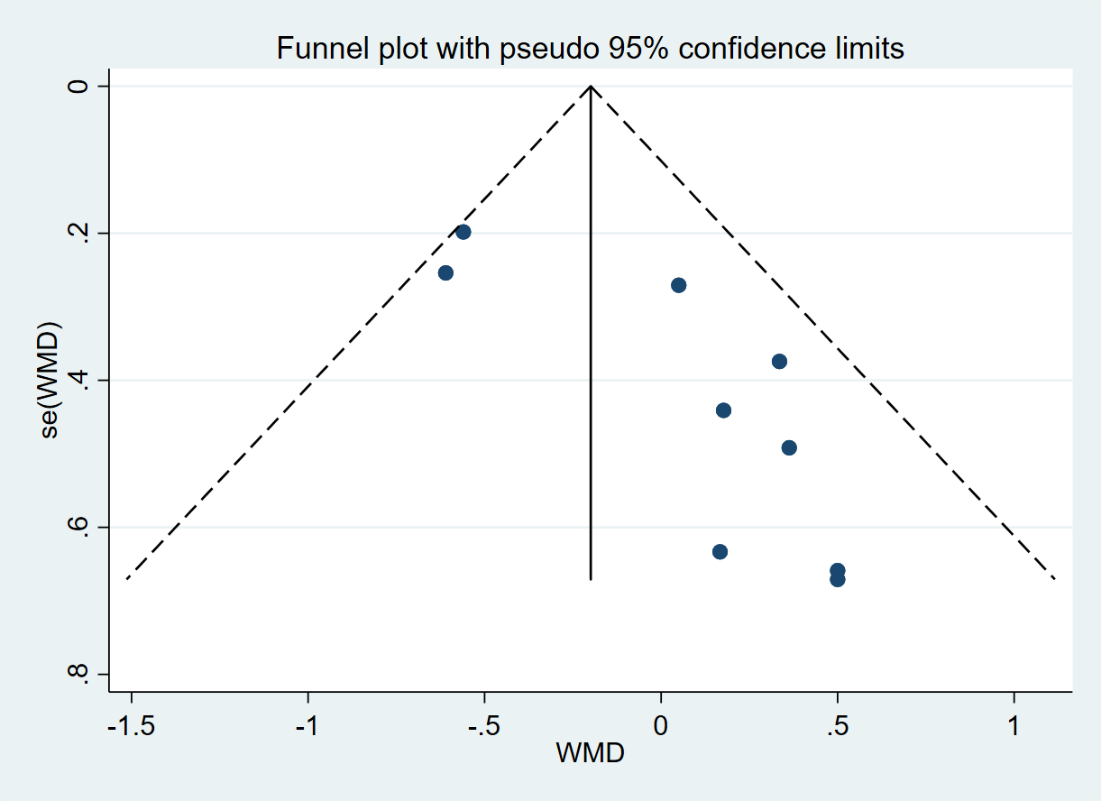


**Supplementary Figure S3.** Funnel plot for the association between nanoparticles and the model group.


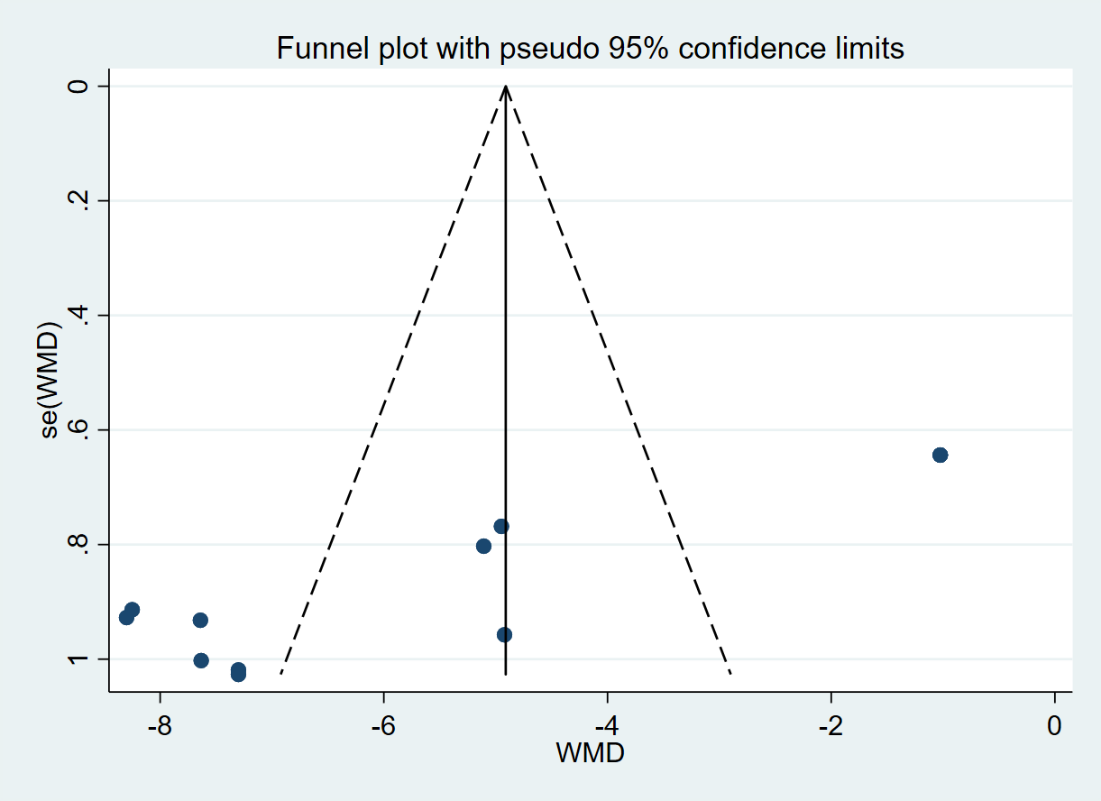

Supplement: Supplementary file 2 — Additional file 2: Supplementary Table S2. Search strategy used in PubMed/ Scopus/ Web of Science / the Cochrane library/ Embase online database. Supplementary Table S3. Important results on the swelling degree of joints and blood biochemical index from studies without meta-analyzed. Supplementary Fig. S1. Quality assessment of included studies using SYRCLE’s risk of bias tool. Supplementary Figure S2. Funnel plot for the association between nanoparticles and allopurinol. Supplementary Figure S3. Funnel plot for the association between nanoparticles and the model group. [file 12891_2023_6186_MOESM2_ESM.docx]
